# 18F-FES PET/CT Improves the Detection of Intraorbital Metastases in Estrogen-Receptor-Positive Breast Cancer: Two Representative Cases and Review of the Literature

**DOI:** 10.3390/tomography8020086

**Published:** 2022-04-07

**Authors:** Sandhya Bodapati, Peter Abraham, Angela Chen, Denise Guilbault, Marin McDonald, Jennifer Matro, Rebecca Shatsky, Sebastian Obrzut

**Affiliations:** 1 College of Osteopathic Medicine of the Pacific, Western University of Health Sciences, 309 E. Second St., Pomona, CA 91766, USA; sandhya.bodapati@westernu.edu; 2 Department of Radiology, University of California San Diego School of Medicine, 200 W Arbor Dr., San Diego, CA 92103, USA; pabraham@health.ucsd.edu (P.A.); awc004@health.ucsd.edu (A.C.); mamcdonald@health.ucsd.edu (M.M.); 3 GE Healthcare, 1053 W Grand Ave., Chicago, IL 60642, USA; denise.guilbault@ge.com; 4 Division of Hematology/Oncology, University of California San Diego School of Medicine, 3855 Health Sciences Dr., La Jolla, CA 92037, USA; jmatro@health.ucsd.edu (J.M.); rshatsky@health.ucsd.edu (R.S.)

**Keywords:** orbital metastases, estrogen-receptor-positive cancer, 18F-fluoroestradiol (18F-FES)

## Abstract

Orbital metastases are a rare but life-altering complication in cancer. Most commonly seen in breast cancer, metastases to the optic nerves or extraocular muscles can have a devastating impact on visual acuity and quality of life. Hormone receptor status plays a central role in metastatic breast cancer treatment, with endocrine therapy often representing first-line therapy in hormone-receptor-positive cancers. Staging and treatment response evaluation with positron emission tomography (PET) computed tomography (CT) imaging with 18F-fluorodeoxyglucose (18F-FDG) is limited by high physiologic uptake in the intracranial and intraorbital compartments. Thus, traditional staging scans with 18F-FDG PET/CT may under-detect intraorbital and intracranial metastatic disease and inaccurately evaluate active metastatic disease burden. In comparison, 18F-fluoroestradiol (18F-FES) is a novel estrogen-receptor-specific PET radiotracer, which more accurately assesses the intracranial and intraorbital compartments in patients with estrogen-receptor-positive (ER+) cancers than 18F-FDG, due to lack of physiologic background activity in these regions. We present two cases of breast cancer patients with orbital metastases confirmed on MR imaging who underwent PET/CT imaging with 18F-FES and 18F-FDG. Multimodality imaging with 18F-FES PET/CT offers higher detection sensitivity of orbital metastases, compared with traditional 18F-FDG PET/CT imaging, and can improve the assessment of treatment response in patients with estrogen-receptor-positive (ER+) cancers.

## 1. Introduction

Visual changes are a rare and devastating complication of metastatic breast cancer (MBC) [1,2]. The incidence of orbital metastases is increasing due to recent improvements in survival in patients with MBC [1,2,3]. Up to one-third of MBC patients are suspected to have orbital micro-metastases, although physical symptoms are rarely reported during their lifetime [3,4]. Rarely (in approximately 11% of cases), visual symptoms are the initial presenting complaint on a diagnosis of MBC [3]. In contrast to metastatic lung cancer, where orbital involvement typically presents early in the metastatic disease course, orbital metastases typically develop late in MBC [3]. Median overall survival in MBC following diagnosis of orbital metastases ranges from 22 to 31 months [3]. Orbital metastases are most commonly diagnosed 4.5–6.5 years after primary breast cancer is diagnosed but can be diagnosed as late as 25 years later [3].

Metastatic breast cancer has a predilection for involvement of the extraocular muscles and surrounding extraconal orbital fat, leading to ocular dysmotility and diplopia [3,4]. Patients with orbital metastases may also experience proptosis, decreased vision, pain, and ptosis [3,4]. A definitive diagnosis of orbital metastases requires surgical or fine-needle aspiration biopsy, both of which portend risks of bleeding, vision loss, diplopia, and infection [4]. Therefore, in the context of a clinical history of breast cancer with high suspicion of metastases, a biopsy is often foregone [4].

Over 60% of breast cancers are estrogen-receptor-positive (ER+), with estrogen being a primary driver of proliferation and cell growth [5]. Furthermore, 95% of invasive lobular carcinomas (ILC) are ER+ [6,7]. Evaluation for whole body metastases is typically performed using positron emission tomography (PET) with computed tomography (CT) imaging with 18F-fluorodeoxyglucose (18F-FDG) radiotracer injection [8]. In 18F-FDG PET/CT molecular imaging, which relies on the Warburg effect, cancer cells preferentially uptake glucose and undergo anaerobic metabolism [9]. Imaging intracranial and extraocular muscle lesions is limited with 18F-FDG PET because of the high baseline physiologic glucose uptake, decreasing detection of metastatic burden, and sometimes leading to inappropriate breast cancer staging [8]. Furthermore, invasive lobular carcinoma (ILC) is characterized by relatively lower proliferation and decreased glycolysis, making it a poor candidate for diagnosis with 18F-FDG PET/CT [5,7].

In comparison, 18F-fluoroestradiol (18F-FES) is a novel radiotracer that targets estrogen receptor expression, providing increased utility in imaging ER+ MBC in areas of high physiologic glucose uptake [6,7,9,10,11,12,13]. In addition, 18F-FES offers a non-invasive method of determining estrogen receptor status for breast cancer metastases in areas that are inaccessible through a biopsy or in patients who are poor surgical candidates [6,13,14,15]. To date, seven clinical trials support the clinical use of 18F-FES as an adjunct to biopsy for confirming recurrence and current estrogen receptor status in MBC [11,12]. Further, 18F-FES allows for imaging ER+ metastases in patients with low-grade malignancies, such as ILC that demonstrate low activity on 18F-FDG PET, and for those with metastasis in locations with high baseline physiologic FDG uptake, as demonstrated by the ocular metastases detected in this case report [11,12,13]. In addition, a positive 18F-FES PET/CT scan can help direct the treatment of orbital metastases.

## 2. Case 1

A 53-year-old female with a history of metastatic ILC presented for staging PET/CT with 18F-FDG. The patient initially presented with stage I, ER+/Her2 negative invasive lobular carcinoma of the left breast in 2007 and underwent chemotherapy with six cycles of cyclophosphamide, methotrexate, and fluorouracil (CMF), lumpectomy, whole breast radiation, and was given adjuvant hormonal therapy with 5 years of tamoxifen. In 2018, a breast MRI demonstrated a large area of clumped, non-mass enhancement in her right lateral breast. Multiple abnormal-appearing right axillary lymph nodes were also identified. MRI-guided biopsy of the right breast revealed grade 2 ILC that was highly ER+/PR+ and Her2 negative.

**Figure 1:**
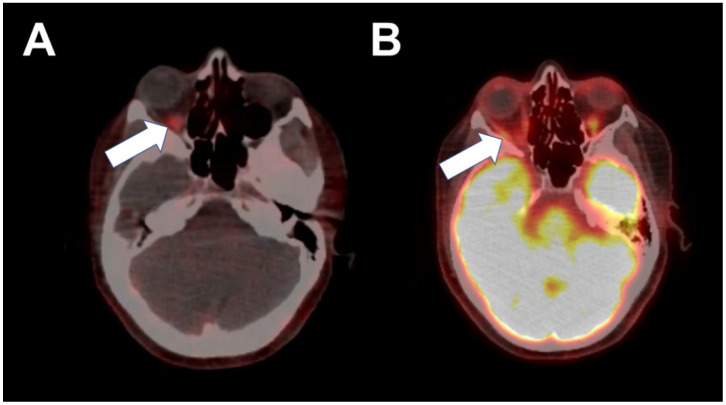
Abnormal intense 18F-FES activity in the right posterior orbit (**A**, arrow), corresponding to known right oculomotor nerve, right medial rectus, and superior aspect of the right inferior rectus. Normal physiologic 18F-FDG activity in the right extraocular muscles (**B**, arrow).

**Figure 2:**
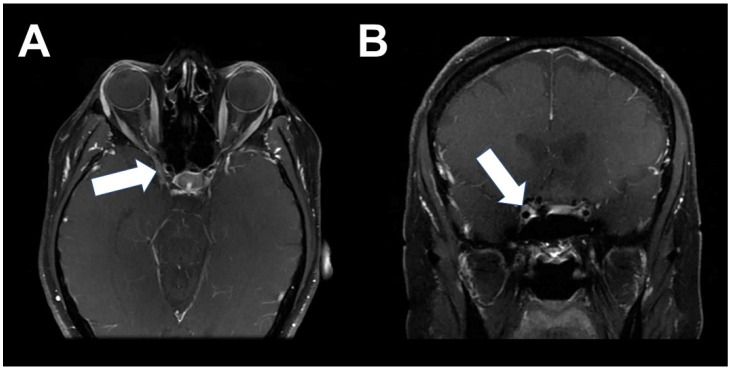
Post-contrast axial T1-weighted fat-saturated magnetic resonance images demonstrate an enhancing tubular structure (**A**,**B**, arrows), with increased posterior extension into the superolateral right cavernous sinus. It courses anteriorly through the right superior orbital fissure and bifurcates in the intraconal space at the orbital apex, before terminating along the lateral aspect of the right medial rectus and superior aspect of the right inferior rectus. Findings are consistent with underlying infiltrative orbital metastatic disease with optic nerve involvement in the setting of known invasive lobular carcinoma.

She underwent chemotherapy with four cycles of docetaxel and cyclophosphamide. After completion of chemotherapy, she presented with one month of progressive vertigo. Brain MRI demonstrated a solitary 3–4 mm enhancing foci located in the subcortical white matter of the right frontal lobe, consistent with metastatic disease. She received stereotactic radiosurgery treatment for the solitary metastasis and had resolution of symptoms and resolution of disease on subsequent brain MRI imaging. She was then started on systemic therapy with fulvestrant and palbociclib. In May 2020, she reported sudden onset of right-sided facial pain, diplopia, as well as increasing right breast pain. The breast MRI revealed the progression of the disease in the right breast. The brain MRI at that time was negative for obvious metastatic disease that might explain her neurologic symptoms, but given the progression of disease in her breast, her systemic treatment was changed to capecitabine. Due to the persistence of symptoms, brain MRI was repeated and this time revealed an enhancing tubular structure involving the optic nerve and the right medial rectus, as well as the superior aspect of the right inferior rectus muscles. She subsequently underwent 18F-FES PET/CT, which showed activity of the posterior orbit, right medial rectus, and right inferior rectus (Figure 1). This activity correlated very closely to the brain MRI findings (Figure 2). Furthermore, there was increased activity in the right breast and right axillary lymph nodes, consistent with the progression of the disease on capecitabine.

## 3. Case 2

A 63-year-old female with a history of invasive lobular carcinoma (ILC), recently diagnosed with osseous metastatic disease in the thoracic spine and peritoneal carcinomatosis presented for staging PET/CT with 18F-FES. She initially presented five years prior with a palpable right breast mass. Ultrasound-guided biopsy demonstrated grade 2 ILC, ER 95%, PR 90%, Her2/neu 0%. A biopsy of an axillary lymph node was positive for metastatic carcinoma. She underwent right lumpectomy and axillary lymph node dissection, which demonstrated 4.0 cm ILC with 15 of 17 ALN positive. Completion mastectomy subsequently revealed 1.5 cm additional ILC.

**Figure 3:**
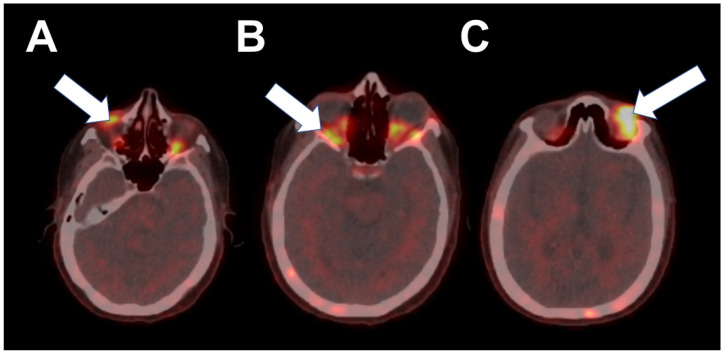
Fused axial PET/CT images demonstrate intense 18F-FES activity in soft tissue in the right inferior (**A**, arrow) and left superior (**C**, arrow) orbits and abnormally increased activity in bilateral extraocular muscles (**B**, arrow).

**Figure 4:**
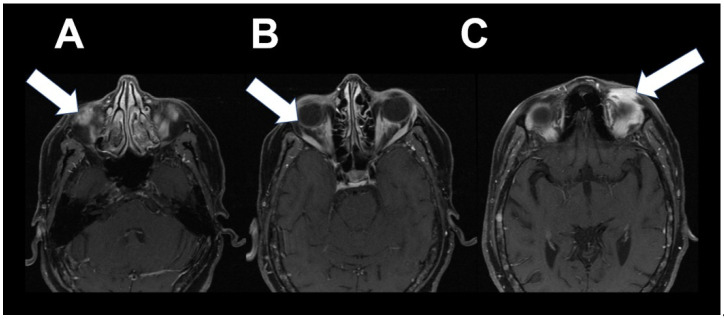
Post-contrast axial T1-weighted fat-saturated magnetic resonance images demonstrate bilateral ill-defined infiltrative enhancement involving the left superior (**C**, arrow) and right inferior (**A**, arrow) preseptal soft tissues extending into the superior extraconal fat, diffuse extraocular muscle enlargement and hyperenhancement (**B**, arrow), consistent with underlying infiltrative orbital metastatic disease in the setting of known invasive lobular carcinoma.

Following adjuvant chemotherapy with dose-dense doxorubicin and cyclophosphamide (AC) and paclitaxel (T) for 12 weeks, she received post-mastectomy radiation to the right chest wall and regional lymph nodes. She was maintained on anastrozole for nearly 5 years until the diagnosis of metastatic disease. One month following discontinuation of anastrozole, 18F-FES PET/CT demonstrated increased activity involving soft tissue nodules in the left superior orbit, right inferior orbit and bilateral extraocular muscles, and numerous foci in the skeleton (Figure 3). Abnormal 18F-FES activity correlated with brain MRI findings, which were consistent with infiltrative orbital metastatic disease (Figure 4). She was started on fulvestrant and palbociclib and was scheduled for radiation to orbital metastases.

## 4. Discussion

Vision is crucial for the quality of life and mediates most of our day-to-day interactions. Loss of vision secondary to under-diagnosis of orbital metastatic disease represents an avoidable devastating outcome of non-specific tumor imaging agents. A more sensitive test is needed for detection of orbital metastases, more accurate staging and treatment planning, as well as early interventions that could spare vision. Chemotherapy and endocrine therapy based on hormone receptor status is the first-line systemic treatment for patients with advanced breast cancer [5]. The mainstay of treatment for orbital metastases, specifically, is external-beam irradiation with a total dose of 20–40 Gy delivered over the course of one to two weeks [3]. Under this regimen of care, up to 60–80% of patients show clinical improvement in local symptoms and vision [3]. Enucleation and other surgical options are mainly considered in palliative cases, for which the procedure is necessary for comfort measures due to intolerable symptoms [3,4]. Ideally, treatment care goals should aim to treat early orbital metastases before they cause diplopia, pain, and other visual symptoms, with the goal of ultimately preventing invasion into the optic nerve or extraocular muscles.

High baseline physiologic uptake of glucose in the brain and extraocular muscles poses a challenge for intracranial and intraorbital imaging with 18F-FDG PET/CT [8]. Strategies for mitigating false-positive results, such as the administration of benzodiazepine muscle relaxants, exist but are not without risks and inconvenience to the patient [8]. As demonstrated in this case series, imaging with 18F-FES is a highly specific and efficacious option for patients with suspected ER+ intraorbital metastases to detect cancer and plan treatment. Two clinical trials demonstrated that 18F-FES PET/CT performs comparably to immunohistochemistry with a 76.6% concordance rate for ER+ tumors, and 100% for estrogen-receptor-negative tumors [11,12]. Based on their results, 18F-FES was found to have a sensitivity of 71.1% in detecting cancer metastases, similar to 18F-FDG, which has a sensitivity of 80% [13]. Most commonly seen in the thoracic area, affinity to estrogen receptor-β may cause false-positive results in non-tumor areas of prior radiation, secondary to high estrogen receptor-β expression in areas of fibrosis [16]. Heterogeneity in tumor hormonal status and recent hormonal therapy with SERMS or SERDs may lead to false-negative results with 18F-FES imaging. Ultimately, clinical suspicion for orbital metastases should direct care management. Quantitative 18F-FES uptake can also be used as a prognostic indicator for response to endocrine therapy, with a residual standard uptake value (SUV) of 1.5 threshold used to identify poor response [13]. In addition, 18F-FES has a six-times higher affinity for estrogen receptor-α than estrogen receptor-β [17]. Since treatment response to hormonal therapy relies on tumor estrogen receptor-α positivity, FES uptake can predict response to endocrine therapy [18].

Usually, 18F-FES is injected intravenously over the course of one to two minutes at a dose of 222 MBq (6 mCi) [11]. PET/CT imaging is recommended 80 min after 18F-FES is administered. Notably, 18F-FES functions by binding to the estrogen receptor [11]. In vitro studies demonstrate binding affinity with a Kd = 0.13 ± 0.02 nM, Bmax = 1901 ± 89 fmol/mg, and IC50 = 0.085 nM [14]. Once injected, the agent is 95% bound to plasma proteins and is ultimately metabolized in the liver and eliminated by the biliary and urinary route [14]. Since estrogen receptor antagonists and modulators can lower estrogen receptor positivity in a tumor site, a washout period is required prior to administration of 18F-FES in patients taking specific estrogen-receptor antagonistic therapies. In patients taking selective estrogen-receptor modulators (SERMs) such as tamoxifen, and estrogen-receptor degraders (SERDs) such as fulvestrant, an 8- and 28-week washout periods, respectively, are warranted [15]. Circulating estrogen- and sex-hormone-binding globulin levels do not affect 18F-FES binding and therefore do not need to be considered in dosing calculations. Imaging with 18F-FES should be completed prior to starting hormonal therapy with these agents such that treatments are not delayed. It should be noted, however, that there is no contraindication or washout period needed for patients on hormonal therapy with aromatase inhibitors.

Given current breast cancer treatment advances, improving median survival while maximizing long-term quality of life has never been more possible. Maintenance of vision is essential to these goals. Clinicians should be aware that 18F-FES could be a useful diagnostic adjunct when evaluating the presence of metastases in breast cancer patients, and should recognize its utility in patients presenting with orbital symptoms. Large-scale studies investigating detection rates of intraorbital metastases with 18F-FES may further extend the role of this radiotracer in breast cancer care, and for imaging ER+ lesions in other areas of high physiologic glucose uptake, such as in the muscles of mastication and salivary glands [8]. Taken together, 18F-FES PET/CT may dramatically affect ER+ cancer care in the coming years, especially in patients with potential intracranial or intraorbital metastases.

## Data Availability

Not applicable.

## References

[1] Antosz Z.S., Walocha J., Porȩba R., Sioma-Markowska U. (2014). Sudden loss of vision due to breast cancer metastasis to the eyeball. Neuroendocr. Lett..

[2] Shields J.A., Shields C.L., Brotman H.K., Carvalho C., Perez N., Eagle R.C. (2001). Cancer metastatic to the orbit: The 2000 Robert M. Curts lecture. Ophthalmic Plast. Reconstr. Surg..

[3] Vlachostergios P.J., Voutsadakis I.A., Papandreou C.N. (2009). Orbital metastasis of breast carcinoma. Breast Cancer Basic Clin. Res..

[4] Ahmad S.M., Esmaeli B. (2007). Metastatic tumors of the orbit and ocular adnexa. Curr. Opin. Ophthalmol..

[5] Burstein H.J. (2020). Systemic Therapy for estrogen receptor–positive, HER2-negative breast cancer. N. Engl. J. Med..

[6] Ulaner G.A., Jhaveri K., Chandarlapaty S., Hatzoglou V., Riedl C.C., Lewis J.S. (2021). Head-to-Head Evaluation of ^18^F-FES and ^18^F-FDG PET/CT in Metastatic Invasive Lobular Breast Cancer. J. Nucl. Med..

[7] Linden H.M., Stekhova S.A., Link J.M., Gralow J.R., Livingston R.B., Ellis G.K., Petra P.H., Peterson L.M., Schubert E.K., Dunnwald L.K. (2006). Quantitative fluoroestradiol positron emission tomography imaging predicts response to endocrine treatment in breast cancer. J. Clin. Oncol..

[8] Purohit B.S., Ailianou A., Dulguerov N., Becker C.D., Ratib O., Becker M. (2014). FDG-PET/CT pitfalls in oncological head and neck imaging. Insights Imaging.

[9] Kurland B.F., Wiggins J.R., Coche A., Fontan C., Bouvet Y., Webner P., Divgi C., Linden H.M. (2020). Whole-Body Characterization of Estrogen Receptor Status in Metastatic Breast Cancer with 16α-18F-Fluoro-17β-Estradiol Positron Emission Tomography: Meta-Analysis and Recommendations for Integration into Clinical Applications. Oncologist.

[10] Gong C., Yang Z., Sun Y., Zhang J., Zheng C., Wang L., Zhang Y., Xue J., Yao Z., Pan H. (2017). A preliminary study of ^18^F-FES PET/CT in predicting metastatic breast cancer in patients receiving docetaxel or fulvestrant with docetaxel. Sci. Rep..

[11] Chae S.Y., Ahn S.H., Kim S.B., Han S., Lee S.H., Oh S.J., Lee S.J., Kim H.J., Ko B.S., Lee J.W. (2019). Diagnostic accuracy and safety of 16α-[ 18 F]fluoro-17β-oestradiol PET-CT for the assessment of oestrogen receptor status in recurrent or metastatic lesions in patients with breast cancer: A prospective cohort study. Lancet Oncol..

[12] Peterson L.M., Kurland B.F., Schubert E.K., Link J.M., Gadi V.K., Specht J.M., Eary J.F., Porter P., Shankar L.K., Mankoff D.A. (2014). A phase 2 study of 16α-[^18^F]-fluoro-17β-estradiol positron emission tomography (FES-PET) as a marker of hormone sensitivity in metastatic breast cancer (MBC). Mol. Imaging Biol..

[13] Chae S.Y., Son H.J., Lee D.Y., Shin E., Oh J.S., Seo S.Y., Baek S., Kim J.Y., Na S.J., Moon D.H. (2020). Comparison of diagnostic sensitivity of [^18^F]fluoroestradiol and [^18^F]fluorodeoxyglucose positron emission tomography/computed tomography for breast cancer recurrence in patients with a history of estrogen receptor-positive primary breast cancer. EJNMMI Res..

[14] Salem K., Kumar M., Kloepping K.C., Michel C.J., Yan Y., Fowler A.M. (2018). Determination of binding affinity of molecular imaging agents for steroid hormone receptors in breast cancer. Am. J. Nucl. Med. Mol. Imaging.

[15] Linden H.M., Kurland B.F., Peterson L.M., Schubert E.K., Gralow J.R., Specht J.M., Ellis G.K., Lawton T.J., Livingston R.B., Petra P.H. (2011). Fluoroestradiol positron emission tomography reveals differences in pharmacodynamics of aromatase inhibitors, tamoxifen, and fulvestrant in patients with metastatic breast cancer. Clin. Cancer Res..

[16] Venema C.M., Mammatas L.H., Schröder C.P., Van Kruchten M., Apollonio G., Glaudemans A.W.J.M., Bongaerts A.H.H., Hoekstra O.S., Verheul H.M.W., Boven E. (2017). Androgen and estrogen receptor imaging in metastatic breast cancer patients as a surrogate for tissue biopsies. J. Nucl. Med..

[17] Seimbille Y., Rousseau J., Bénard F., Morin C., Ali H., Avvakumov G., Hammond G.L., van Lier J.E. (2002). ^18^F-labeled difluoroestradiols: Preparation and preclinical evaluation as estrogen receptor-binding radiopharmaceuticals. Steroids.

[18] Huang B., Omoto Y., Iwase H., Yamashita H., Toyama T., Coombes R.C., Filipovic A., Warner M., Gustafsson J.-Å. (2014). Differential expression of estrogen receptor α, β1, and β2 in lobular and ductal breast cancer. Proc. Natl. Acad. Sci. USA.

